# The Binding of Platinum (II) Complexes to Rabbit Skeletal Muscle G-Actin Induces Conformation Changes

**DOI:** 10.1155/MBD.1995.233

**Published:** 1995

**Authors:** Juan Zou, Fang An, Gang Liu, Kui Wang

**Affiliations:** 1 State Key Laboratory of Coordination Chemistry Nanjing University Nanjing 210093 China; 2 Inorganic Chemistry Department Zhang Jiakou Medical University Zhang Jiakou 075029 China; 3 Inorganic Chemistry Department Beijing Medical University School of Pharmaceutical Sciences Beijing 100083 China

## Abstract

The binding of cis-diamminedichloroplatinum (DDCP) and cis-diaquodiammine platinum (DADP) to rabbit skeletal muscle G-actin and the consequent conformation changes were studied as the function of the Pt/actin molar ratio (R) and time by intrinsic and NPM labeled fluorescence, CD spectra as well as gelfiltration chromatography. The results indicated that the unhydrolyzed DDCP can react with G-actin in presence of Cl^-^ ion. The reaction differs from that of its hydrolysis product DADP in a higher specificity and a lower capacity. Both of them induced exposure of the tryptophane residues and labeled Cys374 and the increase in α-helix content depending on R, but the conformation changes caused by DADP are more significant than DDCP at the same R. These are related to the binding of DADP to groups other than thiols. The rate constants of conformation change suggested that DADP quenched the intrinsic fluorescence more rapid. The temporal change in fluorescence of NPM labeled actin has a biphasic feature: in the first 16 minutes, the fluorescence was quenched, then it recovered slowly, indicating a multi-step reaction including high affinity platinum binding → labeled Cys374 moving to hydrophilic environment → low affinity platinum binding → Cys374-related conformation compacting in sequence.


### THE BINDING OF PLATINUM (II) COMPLEXES TO RABBIT SKELETAL MUSCLE G-ACTIN INDUCES CONFORMATION CHANGES

Juan Zou[1], Fang An[2], Gang Liu[3], and Kui Wang[3*]

[1] State Key Laboratory of Coordination Chemistry, Nanjing University, Nanjing 210093, China
[2] Inorganic Chemistry Department, Zhang Jiakou Medical University, Zhang Jiakou 075029, China
[3] Inorganic Chemistry Department, School of Pharmaceutical Sciences, Beijing Medical University, Beijing 100083, China

### Abstract

The binding of cis-diamminedichloroplatinum (DDCP) and cis-diaquodiammine platinum (DADP) to rabbit skeletal muscle G-actin and the consequent conformation changes were studied as the function of the Pt/actin molar ratio (R) and time by intrinsic and NPM labeled fluorescence, CD spectra as well as gel–filtration chromatography. The results indicated that the unhydrolyzed DDCP can react with G-actin in presence of $Cl^-$ ion. The reaction differs from that of its hydrolysis product DADP in a higher specificity and a lower capacity. Both of them induced exposure of the tryptophane residues and labeled Cys374 and the increase in α-helix content depending on R, but the conformation changes caused by DADP are more significant than DDCP at the same R. These are related to the binding of DADP to groups other than thiols. The rate constants of conformation change suggested that DADP quenched the intrinsic fluorescence more rapid. The temporal change in fluorescence of NPM labeled actin has a biphasic feature: in the first 16 minutes, the fluorescence was quenched, then it recovered slowly, indicating a multi-step reaction including high affinity platinum binding → labeled Cys374 moving to hydrophilic environment → low affinity platinum binding → Cys374-related conformation compacting in sequence.

### Introduction

The interpretation of the anticancer mechanism of cis-diamminedichloroplatinum (DDCP) and the guidelines for designing new antineoplastic platinum complexes have been mostly based on the interaction with DNA. However, the activity and toxicity are both the manifestation of all the events occurring in the course of drug-cell interaction, in which the involvement of non-DNA targets is also important. Evidences accumulated in recent years support that the microfilament system is susceptible to be attacked by platinum complexes[1-5]. The microfilaments are in a dynamic state of interconversion between monomer (G-actin) and polymer (F-actin). We found DDCP and cis-diaquodiammine platinum (DADP) to affect the self-association of G-actin, but in somewhat different ways[6-7]. In order to clarify the mechanism of this action and the difference between DDCP and DADP, the investigation on DDPC (its hydrolysis was inhibited by 0.1 mol/L NaCl) and DADP binding to G-actin has been performed in the present work using spectroscopic and chromatographic techniques.

### Materials and Reagents

### Chemicals

Cis-Diamminedichloroplatinum obtained from Qilu Pharmaceutical Factory. $Na_2ATP$, Sodium azide and

N-(1-pyrenyl) maleimide (NPM) were from Sigma Chemical Co.. All other reagents were of analytical grade and used without further purification, and all solutions were prepared with deionized water. Buffer A (pH 8.0) was composed of 2mol/L Tris, 0.2mmol/L $CaCl_2$, 0.005% $NaN_3$. In the study of DDCP–actin, NaCl was added (0.1mol/L) in order to inhibit hydrolysis, while in the case of DADP–actin, 0.1 mol/L $NaClO_4$ was added instead to keep the ionic strength constant.

### Preparation of DADP Solution

Cis-Diamminedichloroplatinum and silver nitrate (molar ratio 1:1.98) were stirred in the dark at room temperature for 12h. The reaction mixture was chilled and filtered. No $Ag^+$ was found in the filtrate by checking with 0.1mol/L KCl.

### Purification of G-actin

G-actin was extracted from rabbit skeletal muscle as described previously[5]. G-actin concentration was determined according to the absorbance at 290nm with the absorption coefficient $A_{290}^{1mg/ml}$ =0.63[8] and checked with Lowry's method[9]. The level of impurities in the actin is less than 0.5% as determined by SDS –polyacrylamide gel electrophoresis[10].

### Instruments and Methods

### Instruments

All fluorescence measurements were conducted with a Shimadzu RF–540 spectrophotofluorimeter with slits for both excitation and emission set at 5nm. The CD spectra were recorded on a Jasco–500c polarizing spectrophotometer. The assays of platinum were performed with AA–40P Varian atomic absorption spectrophotometer (AAS).

### Gel–filtration Chromatographic Study on the Platinum Binding to G-actin

G-actin was allowed to react with a 22-fold molar excess of DDCP or DADP at 20 °C for 16h and 28h. The samples were then chromatographed on Sephadex G–25 columns equilibrated and eluted with buffer A. The platinum content in each eluted fraction was determined by AAS. The protein content of selected fractions was determined by Lowry's method.

### Study on the Intrinsic Fluorescence Change of G-actin Induced by the Platinum Complexes

The test solutions were prepared by incubating aliquots of G-actin solution (0.4μmol/L) with various amounts of DDCP or DADP for 12h at 4 °C The concentrations of Pt (II) complexes were varied in the range 0–40 μmol/L. The intrinsic fluorescence of samples was measured with $\lambda_{ex}$=286nm and $\lambda_{em}$=340nm at room temperature.

### The Reaction of Platinated G-actin with NPM

The Pt(II)–actin solutions were prepared by incubating G-actin (0.4μmol/L) with various volumes of

DDCP (2.0mmol/L) or DADP (2.0mmol/L) stock solution. After standing at 4 °C for 12h, 25μl 1mmol/L NPM solution was added to each of the solutions, and left overnight at 4 °C. The fluorescence measurements were conducted with $\lambda_{ex}$=342nm and $\lambda_{em}$=375nm.

### The Reaction of NPM Labeled G-actin with DDCP or DADP

G-actin was labeled previously with NPM by Kawasaki's method[11] with a molar ratio 1.0 of NPM to actin. After standing 24h at 4 °C and dialyzing against buffer A to remove the free NPM, the solution was diluted to 0.4 μmol/L. The NPM labeled G-actin solutions were treated with increasing volumes of DDCP or DADP solutions. After standing overnight at 4 °C, the fluorescence was measured with an excitation wavelength of 342nm and an emission wavelength of 375nm.

### CD Measurements

CD spectra were recorded at room temperature. G-actin concentration was 0.4 μmol/L and DDCP or DADP concentrations varied in the range of 0–32μmol/L. The spectra were scanned from 240nm to 190nm and repeated four times at sensitivity 2°cm$^{-1}$.

### The Conformation Change of G-actin Induced by Platinum Binding

Fluorescence measurements were performed in a temperature-controlled cuvette chamber. The cell holder was thermostated at 25 ±0.2°C. The spectra of DDCP–actin and DADP–actin were recorded at the same time. The quenching effect of DDCP or DADP on the intrinsic fluorescence of G-actin was observed at G-actin concentration of 8.35 μmol/L, and the molar ratio of DDCP or DADP to G-actin is 21.8. The change of NPM labeling fluorescence was studied at the G-actin concentration of 0.97μmol/L and a molar ratio of DDCP or DADP to G-actin of 21.0.

### Results

### The Reaction of DDCP or DADP with G-actin

By means of gel–filtration chromatography with Sephadex G–25, the free platinum complexes were separated from the protein, including the platinum bound protein. When G-actin was incubated with DDCP and DADP separately, 5.6mol DDCP and 3.3mol DADP were bound to each mol of G-actin. When the time of incubation was extended to 28h, the average binding numbers in both case were almost unchanged.

Titration curves based on intrinsic fluorescence of G-actin showed that the fluorescence was quenched by DDCP or DADP. An increase of DDCP concentration affected the fluorescence only slightly but, for DADP, the effect was more significant (Figure 1).

Scatchard plots based on the fluorescence titration data are given in Figure 2 and 3. We define $Y=[PtL]/C_{actin}$ , here [PtL] refers to the concentration of the bound Pt and $C_{actin}$ is the concentration of G-actin. A nonlinear regression approach was used for the best fitting and the results indicated that the binding sites might be categorized into two groups both of DDCP and DADP: for DDCP, high affinity binding sites with an average binding constant $K_1=3.0\times10^6$ L/mol, and a number of binding sites $n_1=23$; low affinity binding sites with $K_2=5.1\times10^5$ L/mol, $n_2=38$; for DADP, high affinity binding sites with $K_1=1.7\times10^6$ L/mol, $n_1=40$; low affinity binding sites with $K_2=4.6\times10^5$ L/mol, $n_2=60$.

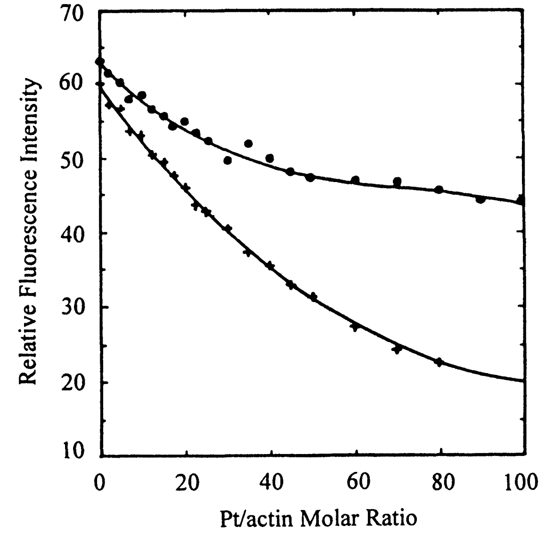

FIGURE 1. The changes of intrinsic fluorescence of G-actin with increasing platinum/actin molar ratio. $C_{actin}$=0.4μmol/L, buffer A. DDCP(•); DADP(+).

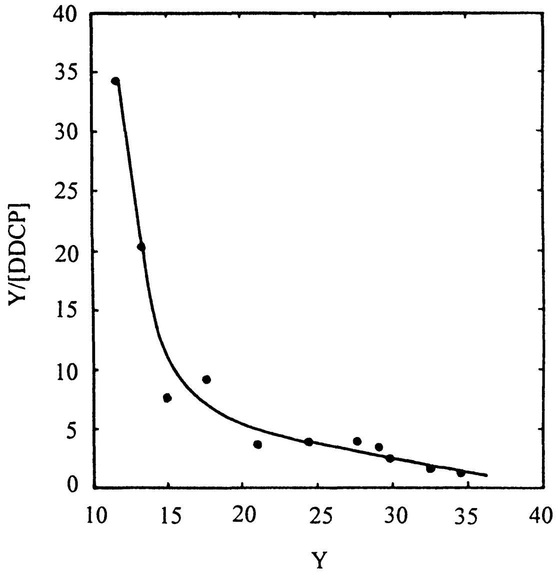

FIGURE 2. Scatchard plot of DDCP binding to G-actin. The experimental conditions see the text. $C_{actin}$=0.4μmol/L, buffer A.

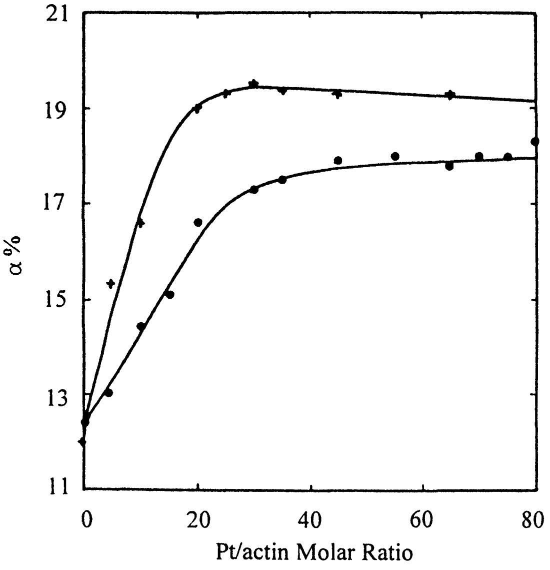

FIGURE 6. α-helix content as a function of platinum/actin molar ratio. $C_{actin}$=0.4μmol/L, buffer A. DDCP (•); DADP(+).

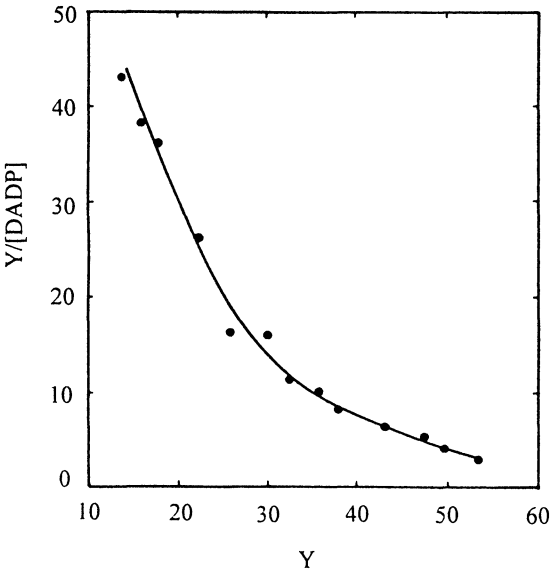

FIGURE 3. Scatchard plot of DADP binding to G-actin. The experimental conditions were described in the text. $C_{actin}$=0.4μmol/L, buffer A.

### The Reaction of DDCP–actin or DADP–actin with NPM

Since NPM binds to Cys374 specifically, it can be deduced that NPM reacts with the Cys374 unoccupied by platinum complexes when G-actin was treated previously with DDCP or DADP. The fluorescence intensity decreased very slightly with increasing DDCP concentration, but the fluorescence intensity decreased dramatically for DADP in the range of Pt/actin less than 20 (Figure 4A and 5A).

### The Reaction of NPM Labeled G-actin with DDCP or DADP.

When labeled G-actin was titrated by DDCP or DADP, the profiles of the change in fluorescence intensity (Figure 4B and 5B) resembles those given in Figure 4A and 5A respectively.

### Cirular Dichroism Measurements

The α-helix content was estimated on the basis of the mean residue ellipticity $\theta_{208}$, as shown on Figure 6. The α-helix content were increased from 12% to 19% (DDCP), from 12% to 18% (DADP) linearly with the increasing R to 35. After then, no changes were observed.

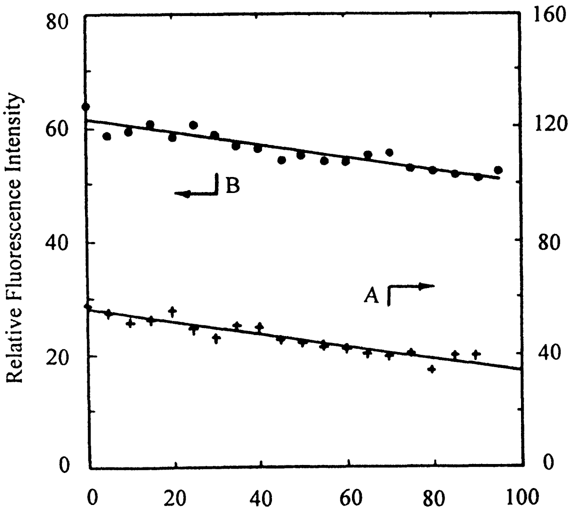

FIGURE 4. The changes of NPM labeled fluorescence as a function of DDCP/actin molar ratio. $C_{actin}$=0.4μmol/L, buffer A. Curve A(+): G-actin was incubated with DDCP at first, then DDCP–actin was labeled by NPM. Curve B (•): G-actin was previously labeled with NPM and free NPM was removed completely, then titrated with DDCP.

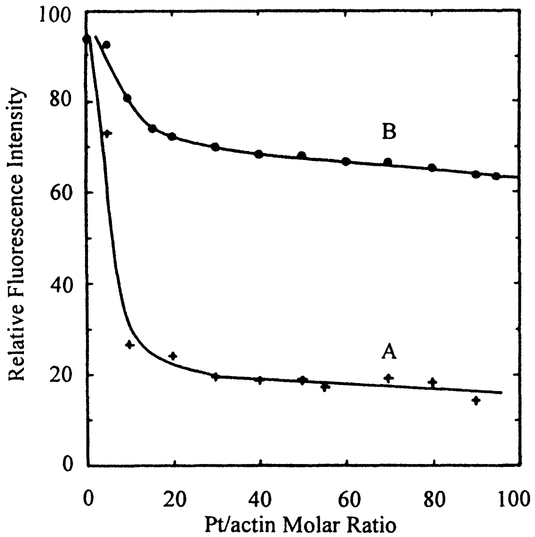

FIGURE 5. The changes of NPM labeled fluorescence as a function of DADP/actin molar ratio. $C_{actin}$=0.4μmol/L, buffer A. Curve A(+): G-actin was incubated with DADP at first, then DADP–actin was labeled by NPM. Curve B (•): G-actin was previously labeled with NPM and free NPM was removed completely, then titrated with DADP.

**The Change of Conformation of G-actin Induced by DDCP or DADP**

DDCP or DADP binding to G-actin was reflected in the temporal change of intrinsic fluorescence as given in Figure 7 and NPM labeling fluorescence (Figure 8). In the presence of DDCP or DADP, the fluorescence intensity of NPM label was quenched sharply in the first 16 min, then it recovered slowly, but a monotonous decreasing of the intrinsic fluorescence was observed in both DDCP and DADP. The pseudo-first order rate constants for the change of fluorescence are summarized in Table 1.

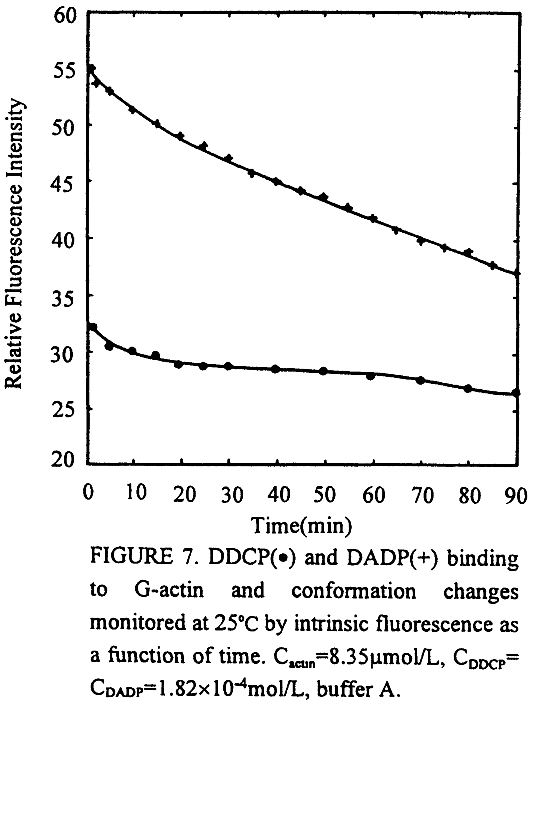

FIGURE 7. DDCP(•) and DADP(+) binding to G-actin and conformation changes monitored at 25°C by intrinsic fluorescence as a function of time. $C_{actin}$=8.35μmol/L, $C_{DDCP}$= $C_{DADP}$=$1.82\times10^{-4}$mol/L, buffer A.

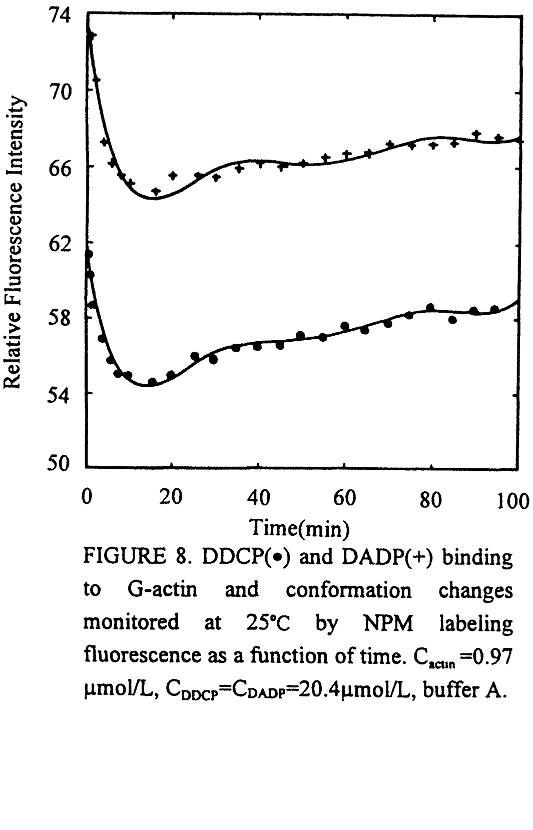

FIGURE 8. DDCP(•) and DADP(+) binding to G-actin and conformation changes monitored at 25°C by NPM labeling fluorescence as a function of time. $C_{actin}$ =0.97 μmol/L, $C_{DDCP}$=$C_{DADP}$=20.4μmol/L, buffer A.

Table 1. The rate contants of conformation change

| | by intrinsic fluorescence | by fluorescence of NPM labeled G-actin | |
|---|---|---|---|
| rate constant ($min^{-1}$) | k | $k_1$ (t<16min) | $k_2$ (t>16min) |
| DDCP | $1.47\times10^{-3}$ | $1.25\times10^{-2}$ | $7.31\times10^{-4}$ |
| DADP | $4.06\times10^{-3}$ | $1.18\times10^{-2}$ | $4.59\times10^{-4}$ |

**Discussion**

There are two possible pathways for DDCP–protein reactions[12], i.e. direct ligand exchange with S-donors of the protein and, indirectly, via the hydrolysis product, DADP. It can be expected that, in the presence of a sufficiently high concentration of $Cl^-$ ion, inhibiting the hydrolysis of DDCP, the reactive species will be the unhydrolyzed DDCP and the solely possible reaction will be the direct displacement of ligand $Cl^-$ by a S-donor. In contrast, in media of lower $Cl^-$ concentration, the reactant is mainly DADP since the complex hydrolyzes ($t_{1/2}$~9h at room temperature)[13]. DADP reacts 10 times faster than DDPC[12], but the difference between DDCP and DADP is not only a kinetic one: the binding capacity and specificity can also play an important role, because DADP tends to react with most of the nucleophilic groups in the protein, including N- and O-donors. It is thus deduced that the binding capacity of DADP is higher than DDCP, and the specificity of binding is less than DDCP. The gel–filtration chromatographic studies gave evidence for the difference in binding capacity. The number of binding sites of DDCP was shown to be only 60% of DADP, as determined on the basis of fluorescence titration.

It is expectable that these differences will cause differences in conformation change. Both DDCP and DADP quenched the intrinsic fluorescence continuously with the increasing concentration (see figure 1), indicating that the tryptophane residues moved to a more hydrophilic environment by binding to high- and low-affinity sites, but since DDCP binds S-donor mainly at lower concentration, the quenching effect became much less at R>20.

The difference between DDCP and DADP became more evident as reflected in NPM-labeled fluorescence, which is an indicator of the microenvironment of Cys-374 in C-terminal. As seen from Figure 4B and 5B, DDCP reduced the NPM fluorescence of NPM-labeled actin monotonously, while the quenching effect of DADP was characterized by a biphasic mode depending on concentration, i. e. a significant drop in fluorescence at lower R and then a very slight reduction over R=20. This might be interpreted by considering that the binding to high-affinity sites caused strong effect to push the C-terminal into the aqueous medium, but the further binding to low-affinity sites caused less effect.

If G-actin was treated previously with DDCP or DADP before labeling with NPM, then the NPM molecules can only react with the thiol groups unoccupied by platinum (Figure 4A and 5A). Comparing the curves of A and B for DDCP or DADP, it can be found that the NPM labeled fluorescence changed in a similar way for either of them, no matter G-actin was incubated with platinum complexes before labeled by NPM or G-actin was labeled by NPM before reacted with platinum complexes. It is reasonable that the reaction of DDCP with thiols is more difficult than that of DADP, since it can be seen that a much lower concentration of DADP is required to reach the equilibrium state than DDCP.

Although the secondary structure of G-actin was affected by both DDCP and DADP, the effect of DDCP was much less pronounced than that of DADP. For lower R values (R<20), the α-helix increased as the result of platinum binding to high-affinity sites. Above this limit, a further increase in α% was observed for DDCP, though with a smaller concentration dependence, but DADP caused no further change. The increase in α-helix content might be related to intermolecular and intramolecular cross-linking. This inference was supported by the result of polyacrylamide gel electrophoresis[7].

According to Figure 8, as the results of DDCP and DADP actions, the kinetic of the Cys374-related conformation changes in a similar way. At the initial stage, a rapid reaction was believed to be the DDCP and DADP binding to sulfur donors of both methionine and cysteine residues; in a second stage, a further slow binding of the platinum complexes to other sites brought about a further conformation change and the molecules became more compact. For DDPC, the pseudo-first order rate constant is 1.6-fold higher than for DADP in this stage (Table 1). The exposure of tryptophane residues proceeded accompanying the C-terminal conformation change. The rate constants revealed that the tryptophane residues were moved to a more opened environment much faster by DADP than DDCP.

In summary, the results reported here indicate that DDCP can react with G-actin directly, but the reaction is different from that of DADP–actin. Since DADP is able to bind to a number of additional sites, especially those other than sulfur donors, the binding of DADP was characterized by a higher capacity and a lower specificity compared to DDCP. As a result, DADP is more potential to affect conformation of G-actin than DDCP, which is the source of the difference between DDCP and DADP in affecting the self-association of G-actin. Hence, it is very important for a platinum complex to hydrolyze at a suitable rate. It is expected that a very slow aquation rate of a platinum complex leads to a less effective binding to DNA and a low-anticancer activity. In contrary, a platinum complex which hydrolyzes too rapidly will react significantly with proteins and cause high toxicity.

**Acknowledgements**

The present work is supported by the State Commission of Science and Technology and National Natural Sciences Foundation of China.

**References**

1. P. Köpf-Maier and S. K. Muhlhausen, Chem.- Biol. Interactions 82, 295-316 (1992).
2. Y.-X. Su et al., J. Mol. Sci. (China) 6, 105-110 (1990).
3. S. K. Aggarwal and A. Sodhi, Cytobiology 7, 366 (1973).
4. S. K. Aggarwal, J. Clin. Hematol. Oncol. 7,760 (1977).
5. J. Zou, H.-Y. Sun, K. Wang, Metal-Based Drugs 2(3), 137 (1995).
6. H.-H. Zeng, Thesis of Doctorate, Inorg. Chem. Dept. of Beijing Medical Univ. 1991.
7. J. Zou, K. Wang, results unpublished.
8. R. Cooke, Biochemistry 14, 3250-3256 (1957).
9. O. H. Lowry, N. J. Rosebrough, A. L. Farr and R. J. Randall, J. Biol. Chem., 193, 265-275 (1951).
10. U. K. Laemmli, Nature 227, 680-685 (1970).
11. Y. Kawasaki, K.Mihashi, H. Janaka and H. Qhnuma, Biochim. Biophys. Acta 446, 166-178 (1976).
12. C. H. Langford and H. B. Gray, Ligand Substitution Processes, W. A. Benjamin, New York (1965).
13. B. J. Corden, Inorganica Chimica Acta 137, 125-130 (1987).

**Received: June 12, 1995 - Accepted: July 5, 1995 - Received camera-ready revised version: July 27, 1995**

